# Effect of Inhaled Medication and Inhalation Technique on Dental Caries in Asthmatic Patients

**DOI:** 10.5812/ircmj.4658

**Published:** 2012-12-06

**Authors:** Marzie Boskabady, Hossein Nematollahi, Mohammad Hossein Boskabady

**Affiliations:** 1 Department of Paediatric Dentistry, School of Dentistry, Mashhad, IR Iran; 2 Applied Physiology Research Center and Department of Physiology, School of Medicine, Mashhad University of Medical Sciences, Mashhad, IR Iran

**Keywords:** Dental Caries, Asthma, Medication, Inhalation Technique

## Abstract

**Background:**

The purpose of this study was to examine the relationship between the type of inhaled medication, length of use, dosing, the inhaler use technique and the severity and duration of disease on the dental caries of asthmatic patients.

**Objectives:**

In the present study, the frequency of dental caries in the asthmatic patients and control group was examined. In addition the possible association of dental caries with disease duration, disease severity, asthma symptoms, chest wheeze, PFT values, and dose of medication and inhalation use technique was evaluated.

**Materials and Methods:**

40 asthmatic patients of both sex (20-30 years old) and 40 age and sex matched controls were studied. In asthmatic patients, the type, dose, duration of medications, the technique of inhaler use and severity and duration of the disease were recorded. The dental health status including DT, MT, FT and DMFT (decay, missing, filling teeth) were examined. In addition, pulmonary function tests (PFTs) were performed for both groups.

**Results:**

All PFT variables in asthmatic patients were significantly lower than those of control group except for FVC (P < 0.001 for all cases). All indices of dental caries in asthmatic group were higher than those of controls which was statistically significant for MT and DMFT (P < 0.005 for both cases). Only FT and DMFT in patients with 11-15 years asthma duration were significantly more than those of 6-10 years (P < 0.05 for both cases). There was no significant correlation between indices of dental caries and disease duration, PFT values; doses of medication or the technique of inhaler use; although the technique of inhaler use was relatively acceptable in all asthmatic patients.

**Conclusions:**

These results showed that dental caries among asthmatic patients was more common than control group which can be due to using inhaler drugs or the technique of inhaler use.

## 1. Background

Asthma is a chronic inflammatory airway disorder and a serious global health problem. According to Global Initiative for Asthma (GINA) report, it is estimated that as many as 300 million people suffer from asthma (1). The prevalence of asthma is 2.8% of the total population in Iranian adult population (2). Several oral health conditions exist among asthmatic patients, especially an increased dental caries risk. The Previous studies regarding dental caries in asthmatic patients showed the conflicting results. Some studies have shown the increased risk of dental caries in asthmatic patient, whereas others failed to show any such association in these patients (3, 4). The results of a recent study showed significantly higher prevalence of caries among asthmatic patients compared to control group as well as a positive correlation between the duration of asthma and the caries indices (5, 6). Therefore, asthma is considered as a risk factor for dental caries (7).

The increased dental caries risk has been attributed to prolong the use of inhaler medications which reduces salivary flow and pH (8). The effect of β2-adrenoceptor agonists on saliva composition and dental caries in asthmatic patients has also been demonstrated (9). It has been also shown that anti-asthma medications including β2-agonists and corticosteroids can have harmful effects on teeth (10). Regardless of the type of inhaler drug on dental caries, the technique of using inhalers may have a crucial role in dental caries. If asthmatic patients do not use their inhaler correctly, the greater amount of drug particles (β2- agonists, corticosteroids, carbohydrate and sugar) will participate in oral cavity leading to higher dental caries in these patients (11). In fact, the study of Khalilzadeh et al., (12) also indicated the reduction of dental caries risk in asthmatic children trained regarding the technique of using inhalers with a spacer.

## 2. Objectives

In the present study, the frequency of dental caries in asthmatic patients and control group was examined. In addition the possible association of dental caries with disease duration, disease severity, asthma symptoms, chest wheeze, PFT values, and dose of medication and inhalation use technique was evaluated.

## 3. Materials and Methods

### 3.1. Studied Groups

The present study was conducted on 40 asthmatic patients (aged 27.48 ± 2.90 years, 28M, 12F) and equal groups of non asthmatic controls (aged 26.65 ± 2.76 years, 28M, 12F). The Asthmatic patients were recruited from the Private Asthma Clinic, Mashhad, IR Iran. All patients had the following criteria: 1) previously diagnosed asthma by a physician and having two or more of the following symptoms: recurrent wheeze, recurrent cough or tightness at rest; wheeze, cough or tightness during night or early morning; wheeze or cough during exercise, 2) having FEV1 and PEF less than 80% of predicted values, 3) had no history or symptoms of cardiovascular or other respiratory diseases that required treatment (excluding the common cold). The studied patients had moderate to severe asthma according to the GINA guideline (1). All asthmatic patients were on active treatment, which in all cases included inhaled Beclamethasone dipropionate (600-1600 µg) or Fluticasone propionate (500 µg), Salmeterol (50 µg) and Salbutamol (400 µg as needed).

The control group was selected from non-asthmatic accompanying subjects of the asthma clinic. They were matched for age, gender, and socioeconomic status with the asthmatic group. The controls were also matched for the family background/diet and other conditions with asthmatic patients and some of control subjects were family members of asthmatic patients. The Asthmatic patients and control subjects were also matched for antibiotic usage. In addition, all groups of asthmatic patients were matched for anti-asthma medication such as antibiotics.The protocol was approved by the Ethics Committee of our institution, and each subject gave informed consent. The study was carried out from July 2009 to July 2010.

### 3.2. Protocol

Information regarding the asthma duration, asthma symptoms, chest wheeze, severity of asthma, type of medication, and the frequency of administration was obtained from medical records of the patients according to the previous studies (1, 2, 13-16) (Table 1). Inhalation use technique of patients was evaluated by a trained nurse. Asthmatics were categorized into mild, moderate and severe categories according to the GINA guideline (1). In addition, the patients were divided to 4 groups with 1-5, 6-10, 11-15 and over 16 years duration of disease. The Asthma symptom score was measured according to Table 2 (2, 14, 15). The degree of wheezing was considered between 0 - 3 as follows: no wheezing = 0, hardly heard wheezing = 1, moderate wheezing = 2, and loud wheezing = 3. The criteria and score for inhalation use technique were: 1) removing the cap of canister and shake the inhaler; not shaking = 0, shaking 1, 2) slow deep expiration; absence of expiration = 0, incomplete expiration = 1, complete expiration = 2, 3) placing the mouthpiece into mouth; not placing the mouthpiece into mouth = 0, placing the mouthpiece into mouth but not closing lips around it = 1, placing the mouthpiece and closing lips around it = 2, 4) slow deep inspiration; absence of inspiration = 0, normal inspiration = 1, fast deep inspiration = 2, slow deep inspiration = 3, 5) pressing the canister to release drug simultaneously with inspiration; before inspiration = 0, a while after inspiration = 1, simultaneously with inspiration = 3 6) holding the breath at the end of inspiration for 10 second; not holding the breath = 0, holding the breath for very short time = 1, holding the breath for around 10 sec = 2 and 7) washing the mouth; not washing the mouth = 0, washing the mouth once = 1, washing the mouth more than once = 3.

**Table 1:** The Score of Respiratory Symptoms

	Score
**Cough**	
None	0
Sleeping well with a little cough	1
Waking once at night	2
Waking most of night	3
**Wheezing**	
No existence during strong exercise	0
Existence only during strong exercise	1
Existence during climbing stairs	2
Existence during ordinary activity	3
**Tightness**	
None	0
Existence in case of exertion	1
Mild tightness without exertion	2
Waking at the morning due to tightness	3
**Total score**	9

**Table 2:** The Score of Respiratory Symptom and Inhaler Use Technique in Asthmatic Patients and Statistical Deference Compared to The Highest Values

Variable	Highest Score	Patients Score, mean ± SD	*P*
**Respiratory symptoms**			
** Cough**	3	0.65 ± 0.12	< 0.001
** Tightness**	3	1.20 ± 0.12	< 0.001
** Wheezing**	3	1.30 ± 0.17	< 0.001
** Total respiratory symptoms**	9	3.43 ± 0.42	< 0.001
** Chest wheeze**	3	1.96 ± 0.15	< 0.001
**Inhaler use technique**			
** Shaking**	0	0.98 ± 0.16	< 0.001
** Slow, deep expiration**	2	1.73 ± 0.45	< 0.001
** Mouth positioning**	2	1.13 ± 0.33	< 0.001
** Slow, deep inspiration**	3	2.75 ± 0.54	< 0.001
** Coincident of inspiration and activation**	3	2.70 ± 0.72	< 0.001
** Breath holding**	2	1.53 ± 0.51	< 0.001
** Mouth Wash**	3	2.13 ± 1.31	< 0.001
** Total score**	15	12.93 ± 2.31	< 0.001

Pulmonary function tests of both groups were measured using a spirometer with a pneumotachograph sensor (Model ST90, Fukuda, Sangyo Co., Ltd. Japan) by a trained nurse. Prior to pulmonary function testing, the required manoeuvre was demonstrated by the operator, and subjects were encouraged and supervised throughout test performance. The Pulmonary function testing was performed using the acceptability standards outlined by the American Thoracic Society (ATS) with subjects in a standing position and wearing nose clips (17). The all tests were carried out between 1600 and 2100 hours. The Lung function tests were performed three times in each subject with an acceptable technique. The highest level for forced vital capacity (FVC), forced expiratory volume in one second (FEV_1_), peak expiratory flow (PEF), maximal mid expiratory flow rate (MMEF), maximal expiratory flow at 75%, 50%, and 25% of the FVC (MEF_75_, MEF_50_, and MEF_25_ respectively) was taken independently from the three curves.

Dental caries of the both groups was examined by a single dentist examiner throughout the study. The examination of subjects was done by using a dental chair under standard illumination. The data was collected by means of a checklist designed to collect information regarding patients' age, gender, and their oral hygiene practices. The DT, MT, FT and DMFT indices were used to assess the dental caries of the subjects. 

### 3.3. Data Analysis

The results were analyzed by using the Statistical Package for Social Sciences version 11.0 (SPSS, Inc., Chicago, IL, USA). The data of PFT values, age, respiratory symptoms, inhalation use technique, dose of medication and asthma duration were expressed as mean ± SD and the other data such as dental caries as mean ± SEM. PFT values, indices of dental caries and age of asthmatic and control groups were compared by using unpaired “t” test and Man Whitney U test. The data of three groups of patients was compared by using one way analysis of variance (ANOVA) with Tukey post test and Kruskal-Wallis test. The respiratory symptoms and the score of inhalation technique were compared with their high score by using one sample t test. The correlation between the dose of medication, PFT values, inhalation use technique, asthma duration, severity of disease with indices of dental caries was tested by using Pearson Correlation Coefficient. The Spearman correlation coefficient was also used to determine the correlation between the inhalation use technique and caries status. The significance was accepted at P < 0.05.

## 4. Results

### 4.1. The Score of Respiratory Symptom and Inhaler Use Technique in Asthmatic Patients

All respiratory symptoms were significantly lower than the highest score (P < 0.001 for all cases, Table 2). In addition, all criteria for inhalation use technique in asthmatic patients were significantly lower than the best score (P < 0.001 for all cases, Table 2). 

### 4.2. The Comparison of PFT Values and Indices of Dental Caries Between Asthmatics and Normal Subjects

**Figure 1:**
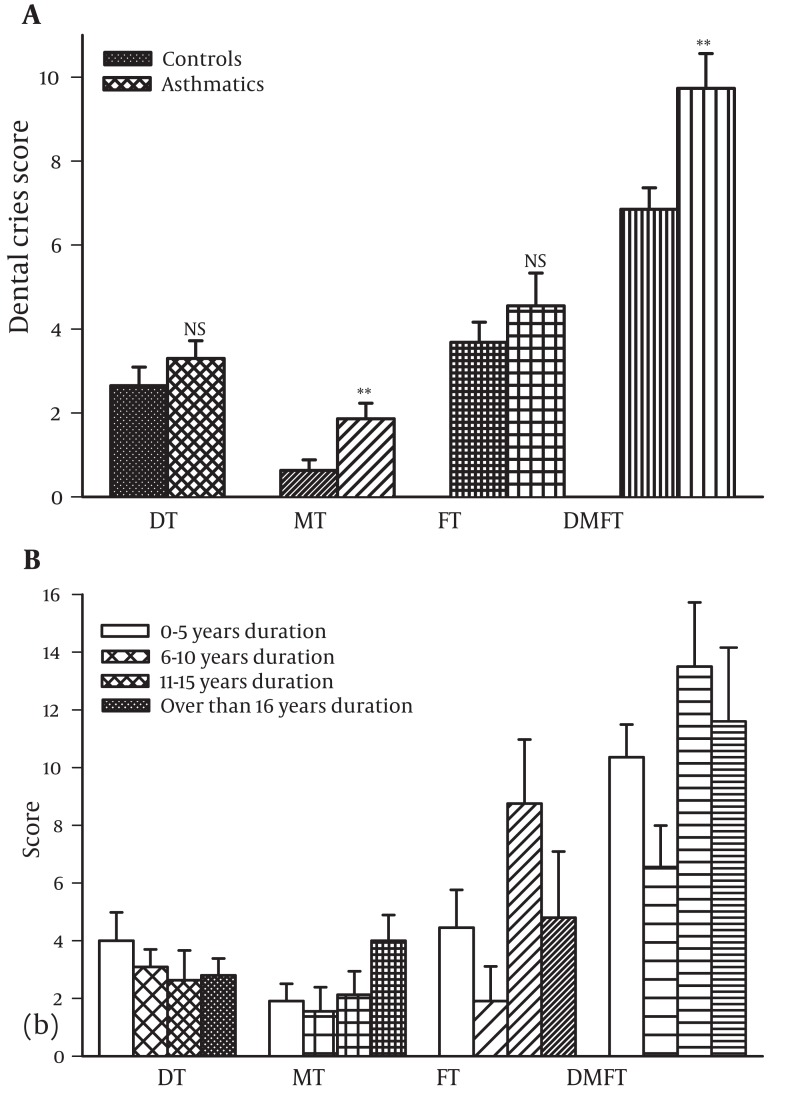
Comparison of indices and dental caries between asthmatic (fine filled bars) and control subjects (medium filled bars) (a) between asthmatic aged 1-5 (open bars), 6-10 (coarse filled bars), 11-15 (medium filled bars) and more than 16 years (fine filled bars) duration of disease (b) . The data were presented as mean±SD and values of PFTs were quoted as percentage predicted. The statistical difference in different parameter between asthmatic and control subjects: NS; non-significant difference, **; P < 0.005, ***; P < 0.001. There was no significant difference in indices and dental caries between 4 groups of asthmatic patients.

All PFT variables were abnormally low in studied asthmatic patients (less than 80% predicted values) and were significantly lower than control group except FVC (P < 0.001 for all cases). In addition all indices of dental caries were higher in asthmatic patients than control group which were statistically significant for MT and DMFT (P < 0.005 for both cases, Figure 1a).

### 4.3. Dental Caries in Asthmatics With Different Disease Severity and Disease Duration

**Figure 2:**
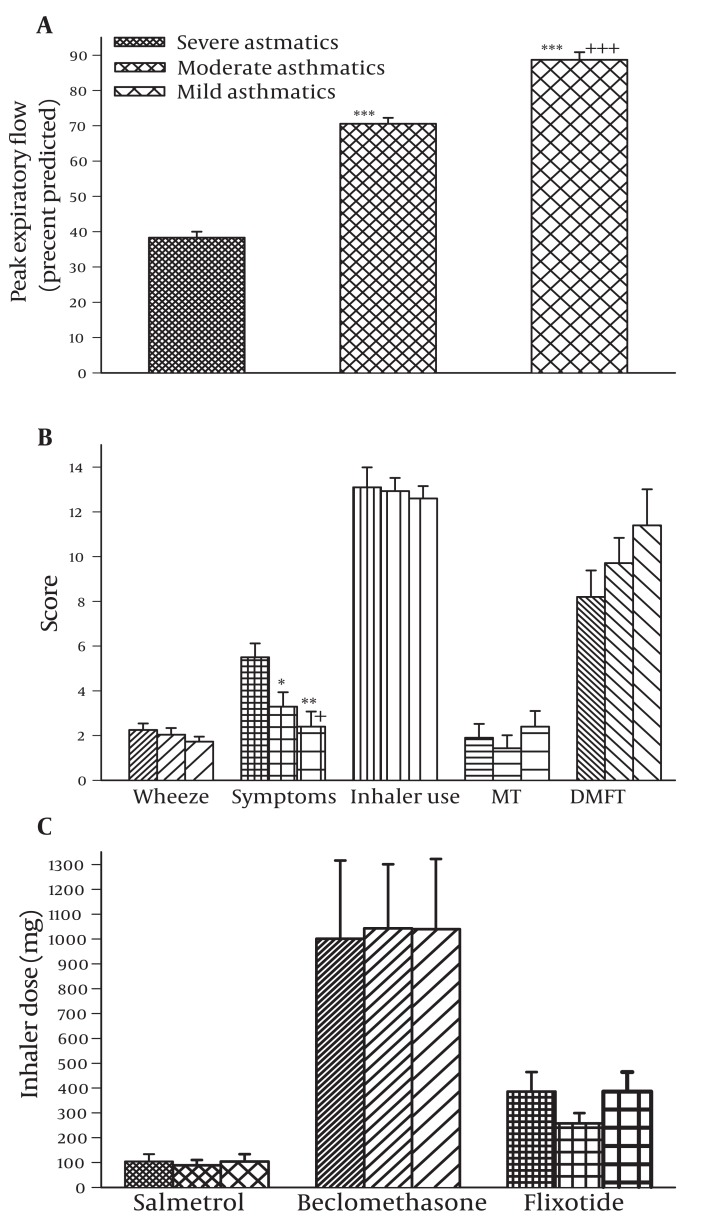
Comparison of PEF (peak expiratory flow, a), respirators symptoms (b) and the dose of inhaled medication (c) between severe (fine filled bars), moderate (medium filled bars) and mild (coarse filled bars) asthmatic patients. The data were presented as mean ± SD and values of PFF were quoted as percentage predicted. The statistical difference in different parameter between severe vs moderate and mild asthmatic patients: *; P < 0.05, **; P < 0.01, ***; P < 0.001. Statistical difference in different parameter between moderate and mild asthmatic patients: +; P < 0.05, +++; P < 0.001.

Peak expiatory flow in severe asthmatics was significantly less than moderate and mild asthmatics and that of moderate patients was less than mild asthmatics (P < 0.001 for all cases, Figure. 2a). In addition, asthma symptoms and chest wheeze in severe asthmatics were higher than moderate and mild asthmatics and those of moderate patients was higher than mild asthmatics patients (only significant for symptoms, P < 0.05 to P < 0.01, Figure 2b). However, there was no significant difference in dental caries score, medication dose or inhalation use technique between three groups of the patients (Figures 2b and c). Regarding the duration of disease there was no significant difference in dental caries score between four groups of the patients (Figure 1b).

### 4.4. The correlations Between Dental Caries Scores With Different Parameters of Asthmatic Patients

There was no significant correlation between dental caries score and disease duration, disease severity, asthma symptoms, chest wheeze, PFT values, dose of medication or inhalation use technique score.

## 5. Discussion

In the present study, the dental caries in young adult asthmatics (aged 20-30 years) compared to a matched non-asthmatic control group was studied. In addition, the role of asthma medication, the technique of inhaler use, the severity and duration of the disease on dental carries was evaluated in asthmatics. The results showed higher prevalence of dental caries among asthmatic patients compared to controls. However, there was no significant difference in dental carries in patients with different severity or different duration of the disease. In addition, there was no significant correlation between dental caries and the dose of medication or the technique of inhaler use. However, the doses of medications among patients of different severity were almost similar. Although the score of inhalation technique use were significantly lower than the best technique, almost all studied patients were able to use their inhaler drugs more or less correctly.

The DMFT score was significantly higher in asthmatic patients compared to control group which was similar to other studies (8, 18, 19). However, the findings of the present study differ from some other studies reporting no significant difference between mean DMFT scores of asthmatics and controls (3, 4, 20). This discrepancy could be due to the age of the patients because in the present study, the young adults' patients were studied but in all studies showing the absence of a difference in dental caries between asthmatics and control, the children asthmatics were evaluated. These results can indicate that the time period of inhalation therapy could be a determinant factor causing dental caries. The other reasons for this discrepancy could be the type of medications used by the patients, the socioeconomic status of the patients and specially the technique of inhaler use which should be clarified in further studies.

The possible cause of an increase in caries prevalence among asthmatic patients could be due to the disease itself including the severity and/or duration of the disease or due to their medications including the type, dose and duration of medications or the technique of inhaler use. The results of the present study did not show any difference in dental caries between patients with different severity or duration of the disease which was in line with previously performed studies indicating that the disease itself does not affect dental caries (5).

The previous studies showed a relationship between the type of drugs specially use of ß_2_ agonists and dental carries (9). In addition, the effect of inhaler corticosteroid on dental caries was also documented (19, 21). However, the results of the present study did not show any correlation between the dose and duration of the medication and dental caries in asthmatic patients. The reason of these findings is perhaps the similar therapeutic regimen among the studied patients. Although the previous studies showed increased precipitation of inhaled drugs in mouth cavity (11) and suggested increased dental caries in patients using their inhaler drugs (12), the results of the present study did not show any significant correlation between dental caries score and the score of the technique of inhaler use. The possible explanation for these results may be due to the acceptable technique of inhaler use among all the studied asthmatic patients. Some previous studies emphasized on the role of technique of inhaler use on dental caries in asthmatic patients (12, 22). However, both of these two studies were conducted on asthmatic children with perhaps shorter duration of using their inhaler drugs. Therefore, the role of the technique of inhaler use in dental caries which could have an important role in this phenomenon should be investigated in patients with different technique of inhaler use in further studies. In conclusion, the findings of this study showed increased dental caries in young adult asthmatics which is perhaps due to their inhaler medications or the technique of inhaler use.
